# Folic acid supplementation before and during pregnancy in the Newborn Epigenetics STudy (NEST)

**DOI:** 10.1186/1471-2458-11-46

**Published:** 2011-01-21

**Authors:** Cathrine Hoyo, Amy P Murtha, Joellen M Schildkraut, Michele R Forman, Brian Calingaert, Wendy Demark-Wahnefried, Joanne Kurtzberg, Randy L Jirtle, Susan K Murphy

**Affiliations:** 1 Department of Community and Family Medicine and Program of Cancer Detection, Prevention and Control, Duke University, PO Box 104006, Durham, NC 27710, USA; 2 Department of Obstetrics and Gynecology, Division of Maternal-Fetal Medicine, Duke University, 4022 Hospital South, Durham, NC 27710, USA; 3 Department of Epidemiology, MD Anderson Cancer Center, 1515 Holcombe Blvd., Unit 1340, Houston, TX 77030-4009, USA; 4 Department of Behavioral Science, MD Anderson Cancer Center, 1515 Holcombe Blvd., Unit 1340, Houston, TX 77030-4009, USA; 5 Department of Pathology and Carolinas Cord Blood Banking Project, Duke University, 1430 N. Pavilion Bldg, Durham, NC 27705, USA; 6 Department of Radiation Oncology, 139 Env Safety Bldg., Durham, NC 27710, USA; 7 Department of Obstetrics and Gynecology, Division of Gynecologic Oncology, Department of Pathology Duke University, 226 B Wing LSRC Research Drive Durham, NC 27708, USA

## Abstract

**Background:**

Folic acid (FA) added to foods during fortification is 70-85% bioavailable compared to 50% of folate occurring naturally in foods. Thus, if FA supplements also are taken during pregnancy, both mother and fetus can be exposed to FA exceeding the Institute of Medicine's recommended tolerable upper limit (TUL) of 1,000 micrograms per day (μg/d) for adult pregnant women. The primary objective is to estimate the proportion of women taking folic acid (FA) doses exceeding the TUL before and during pregnancy, and to identify correlates of high FA use.

**Methods:**

During 2005-2008, pre-pregnancy and pregnancy-related data on dietary supplementation were obtained by interviewing 539 pregnant women enrolled at two obstetrics-care facilities in Durham County, North Carolina.

**Results:**

Before pregnancy, 51% of women reported FA supplementation and 66% reported this supplementation during pregnancy. Before pregnancy, 11.9% (95% CI = 9.2%-14.6%) of women reported supplementation with FA doses above the TUL of 1,000 μg/day, and a similar proportion reported this intake prenatally. Before pregnancy, Caucasian women were more likely to take FA doses above the TUL (OR = 2.99; 95% = 1.28-7.00), compared to African American women, while women with chronic conditions were less likely to take FA doses above the TUL (OR = 0.48; 95%CI = 0.21-0.97). Compared to African American women, Caucasian women were also more likely to report FA intake in doses exceeding the TUL during pregnancy (OR = 5.09; 95%CI = 2.07-12.49).

**Conclusions:**

Fifty-one percent of women reported some FA intake before and 66% during pregnancy, respectively, and more than one in ten women took FA supplements in doses that exceeded the TUL. Caucasian women were more likely to report high FA intake. A study is ongoing to identify possible genetic and non-genotoxic effects of these high doses.

## Background

Previous studies have demonstrated that daily folic acid (FA) supplementation during preconception lowers the risk of neural tube defects [1-4] and other adverse pregnancy outcomes such as low birth weight [5]. Because of this beneficial effect with no apparent adverse health outcomes in pregnancy, FA supplementation of approximately 400 micrograms per day (μg/d) from fortified foods, supplements, or both, was recommended for all women at risk of pregnancy [3,6]. To meet this recommendation, women take over-the-counter multivitamin supplements containing approximately 400 μg/d of FA [7]. Since clinical trials data showed that the full benefit of FA was conferred prior to conception and the national unintended pregnancy rate in the US is 49% [8], a more effective vehicle to reach women was pursued. In 1996, the Food and Drug Administration (FDA) approved population-wide fortification of milled grain at 140 μg FA/100 grams [9] to deliver an additional 100 μg/d of FA to the average adult diet [10]. However, total folate intake exceeding the recommended limit has been reported among women of child-bearing age [11,12].

Folic acid added to foods during fortification is 70-85% bioavailable compared to 50% of folate occurring naturally in foods [6,13]. Thus, if FA supplements also are taken during pregnancy, both mother and fetus can be exposed to FA exceeding the Institute of Medicine's [6] recommended tolerable upper limit (TUL) of 1,000 μg/d for adult pregnant women. While folate is essential for supplying methyl groups for nucleotide synthesis and DNA replication, the effects of high FA doses are unknown. What is known is that since fortification began, American sero-surveillance data indicate that circulating folate concentrations in serum and erythrocytes have increased 50-100% in the general population [14,15]. While FA supplementation use before and during pregnancy has been monitored in numerous populations [16-21], the extent of high FA use in pregnant women is unknown. Herein, we contribute to the monitoring effort by estimating the proportion of pregnant women reporting intake of FA doses exceeding the TUL, and determining whether socio-demographic and lifestyle factors are associated with high use within the Newborn Epigenetics STudy (NEST).

## Methods

### Study participants

Study participants were recruited as part of the ongoing Newborn Epigenetics STudy (NEST), a prospective study of women and their children. NEST was designed to identify early exposures associated with stable epigenetic alterations in infants that may alter chronic disease susceptibility later in life. Trained recruiter/interviewers identified eligible participants by reviewing prenatal care appointment logs in Duke's Division of Maternal and Fetal Medicine at the beginning of each week, and eligible participants were invited to participate. Women were eligible if they were aged 18 years and older, pregnant and spoke English. To ensure access to labor and birth outcomes data, we excluded women who planned to receive obstetric care outside the Duke Obstetrics or Durham Regional Hospitals. The catchment area for Duke Maternal Fetal Medicine prenatal care clinic largely includes three contiguous counties in central North Carolina (NC); Durham, Orange and Wake. Women who met eligibility criteria were either consented and interviewed in-person, in consultation rooms during the visit, or were given the questionnaire to self-administer and mail back to the study office. If completed questionnaires were not received in the mail by the subsequent visit, another questionnaire was administered during a prenatal care visit. Interviewer-administered questionnaires took approximately 15 minutes to complete. At the end of the interview, all women were asked to provide contact information that included names, address and at least three telephone numbers to facilitate future contact. Because *in utero *exposure to cigarette smoke is associated with poor birth outcomes and may predict high FA use [17,22], we targeted smokers to the extent possible, identifying them through medical records.

Gestational age at enrollment ranged from 19 to 42 weeks (mean = 38.1; sd = 2.5 weeks). By June 2008, 838 eligible women had been identified and 601 were enrolled (response rate of 71.7%). Most enrollees (98.2%) were successfully followed to delivery (n = 590). Children from these pregnancies are being followed to collect growth data once every two years. These analyses include 539 participants with complete questionnaire and medical records data. The study protocol was approved by the Duke University Institutional Review Board.

### Data Collection

Domains for which information pertaining to the year before and the year during pregnancy was solicited included the following; demographic characteristics, health status, reproductive factors, lifestyle factors such as tobacco and alcohol use, anthropometric measurements before pregnancy and dietary supplementation. Although not used in the current analyses, diet was assessed using the 24-hour dietary recall (Nutritional Data Systems for Research, University of Minnesota). Medical records were also abstracted to obtain information on maternal morbidity and use of over-the-counter and prescribed medication. At delivery, we collected data related to labor, umbilical cord blood and birth outcomes, including birth weight.

#### Folic acid supplementation

To ascertain FA intake, participants were shown a list of dietary supplements that included multivitamins, multivitamins with additives such as herbs, FA/folacin, vitamin B6 (pyridoxine), vitamin B12 (cobalamin), and "other," and asked for Yes-No responses to the questions "In the 12 months before pregnancy, did you take [dietary supplement]?". To obtain folic acid intake during pregnancy, women were asked to respond to the question "Since you found out you were pregnant, did you take [dietary supplement]?" Supplement users were asked the brand name, the frequency of intake and trimester when intake started, if intake started during pregnancy. Study participants were unaware of the study hypothesis. The frequency and dose of FA intake before and during pregnancy was converted into daily μg/day FA intake using dosage information provided on the packet. Where specific brands were not recalled, a conservative value of 400 μg was assigned.

### Statistical analyses

Women were categorized as non-users (no supplementation), users within recommended range (intake ≤1,000 μg/d), and users exceeding the TUL for adults (intake >1,000 μg/d). A dose of ≤1,000 μg/d was assigned to women reporting "over-the-counter daily multivitamin" as these supplements provide ~400 μg/d of FA. Women taking "additional FA" providing ~800 μg/d of FA, and those reporting intake of "prenatal vitamins only" which provide ~600-1,000 μg/d of FA, were also included in the category of users within the recommended range. Doses exceeding 1,000 μg/d were assigned to women who reported intake of a combination of "prenatal vitamins" including those prescribed (~600-1,000 μg/d) and "over-the-counter multivitamins" (~400 μg/d) of FA, and/or multivitamins with "additional FA" (~400 μg/d). Total FA intake ranged from 0 to >1,600 μg/d.

FA supplementation before and during pregnancy was examined in relation to factors previously identified to predict *any *FA use including ethnicity [23], advanced maternal age [24], socioeconomic indicators (education, marital status and public or private health insurance) [17,24] reproductive factors such as parity [17], use of cigarettes [17] and alcoholic or caffeinated beverages. We also evaluated FA supplementation in relation to factors not previously described, including participants' self-reported health indicators, a history of chronic illness (asthma, hypertension, depression, cardiovascular diseases, allergies, migraines, epilepsy, anxiety, cancer, gestational diabetes) and the use and type of assisted reproductive technology (ART) used in infertility treatments since infertility is also associated with FA use [17].

We estimated the proportions and 95% confidence intervals (95% CIs) of women reporting FA use exceeding the TUL, and examined correlates of use that could be used to target women for further study and possible intervention. Unconditional logistic regression models were used to estimate odds ratios (ORs) and 95% CIs for the association between each correlate and FA supplementation. Non-users were compared to users within recommended range (≤1,000 μg/d), and users exceeding the TUL (>1,000 μg/d), separately for use before and during pregnancy. Cigarette smoking was included in all statistical models except when we stratified by cigarette smoking. Independent effects were evaluated by mutual adjustment in parsimonious models. All statistical analyses were conducted in SAS v9.03 (SAS Institute Inc., Cary, NC).

## Results

Characteristics presented in **Table **1 suggest that study participants were similar to the general pregnant population in the three-county region of NC with respect to age, marital status and education of African Americans and Whites [25]. However, study participants were more likely to smoke or be exposed to environmental tobacco smoke than those in the general three-county population.

**Table 1:** Characteristics of New Born Epigenetics STudy (NEST) Study Participants

Maternal Characteristic	(n = 539)	
	Number	(%)
**Maternal age at beginning of pregnancy**		
<25 years	166	(30.8)
25-34 years	261	(48.4)
35-39	91	(16.9)
40+	20	(3.7)
Missing	1	(0.2)
**Maternal ethnicity**		
African American	258	(47.9)
Caucasian	246	(45.6)
Asian/Native American/Latino	26	(4.8)
Missing	9	(1.7)
**Maternal marital status**		
Never married	154	(28.6)
Married	241	(44.7)
Living with partner	99	(18.4)
Divorced/separated	44	(8.2)
Missing	1	(0.2)
**Maternal education**		
Less than high school	63	(11.7)
High school graduate/GED	146	(27.1)
Some college	145	(27.1)
College graduate	98	(18.2)
Graduate education	86	(16.0)
Missing	1	(0.2)
**Type of health insurance**		
Public (e.g., Medicaid, SCHIP)	250	(46.4)
Private	174	(32.3)
Missing	115	(21.3)
**Parity**		
One	173	(32.1)
Two	113	(21.0)
Three or more	241	(44.7)
Missing	12	(2.2)
**Cigarette smoking at interview**		
Non-smokers	425	(78.9)
Smoker during pregnancy	113	(21.0)
Missing	1	(0.1)
**Exposure to secondhand smoke during index pregnancy**		
Yes	158	(29.3)
No	375	(69.6)
Missing	6	(1.1)
**Pre-pregnancy body size**		
<18 kg/m2	6	(1.1)
≥18 g/m2 - <25 kg/m2	164	(30.4)
≥25 kg/m2 - <30 kg/m2	96	(17.8)
≥30 kg/m2	206	(38.2)
Missing	66	(12.2)
**Therapeutic interventions**		
Hormonal contraceptive us up to one year before interview	135	(25.1)
Use of assisted reproductive technology for index pregnancy	17	(3.2)
**Co-morbid conditions at interview**		
None	341	(63.3)
Gestational diabetes	42	(7.8)
Type 1 or Type 2 diabetes	27	(5.0)
Asthma	88	(16.3)
*Other	39	(7.2)
**Any chronic disease	196	(36.4)
**Folic acid intake before pregnancy**		
None	265	(49.2)
Up to 1,000 μg/day	209	(38.8)
More than 1,000 μg/day	64	(11.9)
Missing	1	(0.2)
**Folic acid intake during pregnancy**		
None	182	(33.8)
Up to 1,000 μg/day	295	(54.7)
More than 1,000 μg/day	61	(11.3)
Missing	1	(0.2)

*Other includes hypertension, depression, heart diseases, allergies, migraine headaches, epilepsy, anxiety, treated for cancer

**Any chronic disease includes gestational diabetes, asthma, hypertension, depression, diabetes mellitus, heart diseases, allergies, migraine headaches, epilepsy, anxiety, treated for cancer

### FA supplementation before and during pregnancy

Overall, 51% (95%CI = 47-55%) of women reported some FA use before pregnancy. Recommended FA doses (≤1,000 μg/d) taken either as a multivitamin or a single dietary supplement were reported by 209/539 or 39% (95%CI = 35%-43%), and intake of doses exceeding the TUL for adults (>1,000 μg/d) was reported by 12% (95%CI = 9%-15%) of women before pregnancy **[Table **1**]**. During pregnancy, some FA use was reported by 66% (95%CI = 62%-70%) of women. FA intake within recommended doses of ≤1,000 μg/d was reported by 295/539 or 55% (95%CI = 51-59%), and intake of doses exceeding the TUL for adults of >1,000 μg/d was reported by 11% (95%CI = 8%-14%) of women. Because we targeted smokers, we also evaluated whether FA use exceeding the TUL varied by smoking status. We found no statistically significant differences in FA use by smoking status as 6% (95%CI = 2%-11%) of smokers and 13% (95%CI = 10%-17%) of non-smokers reported FA intake ≥1,000 μg/d before pregnancy (p-value = 0.11) (data not shown). During pregnancy, the proportion of women reporting FA use exceeding TUL for adults was also similar in smokers 12% (95%CI = 6%-17%) and non-smokers 11% (95%CI = 8%-14%) (p-value = 0.81).

### Correlates of dietary folic acid supplementation

#### FA supplementation before pregnancy

Compared to non-users, FA intake exceeding 1,000 μg/d before pregnancy was reported more frequently by women who were aged >35 years (OR = 5.5, 95% CI = 2.3-13.2), married or living with partner (OR = 3.5, 95%CI = 1.9-6.6), privately insured (OR = 4.7,95%CI = 2.5-8.9), Caucasian (OR = 3.6, 95%CI = 2.0-6.5), and women with a college or higher education (OR = 8.5,95%CI = 2.0-36.3). Smokers (OR = 0.4, 95%CI = 0.2-0.8), those exposed to second hand smoke (OR = 0.4, 95%CI = 0.2-0.9) and those reporting any chronic disease (OR = 0.4, 95%CI = 0.2-0.8), were less likely to report FA intake exceeding the TUL. Mutual adjustment of these factors revealed some attenuation of these effects as FA intake exceeding the TUL remained significantly more likely to be reported by Caucasian women (adjusted OR = 3.0, 95% CI = 1.3-7.0), and less likely by women reporting at least one chronic condition during pregnancy (adjusted OR = 0.5, 95% CI = 0.2-1.0). The direction of the association between high FA intake and advanced maternal age (adjusted OR = 2.2, 95% CI = 0.8-6.4), being married or living with a partner (adjusted OR = 1.9, 95% CI = 0.9-4.0), and having a college or higher education (OR = 7.6, 95% CI = 0.9-65.2) were maintained in multivariate analyses, although the magnitude of associations was attenuated **[Table **2**]**. The direction and magnitude of these associations was similar among FA users exceeding the TUL and those whose intake was within recommended doses, and among smokers.

**Table 2:** *Adjusted odds ratios (ORs) and 95% confidence intervals for the association between maternal characteristics and folic acid supplement before and during pregnancy

	FOLIC-ACID USE BEFORE PREGNANCY					FOLIC-ACID USE DURING PREGNANCY				
Characteristic	None n = 265	<1,000 μg/day n = 209	≥ 1,000 μg/day n = 64	<1,000 μg/day OR(95%CI)	≥ 1,000 μg/day OR(95%CI)	None n = 182	<1,000 μg/day n = 295	≥ 1,000 μg/day n = 62	<1,000 μg/day OR(95%CI)	≥ 1,000 μg/day OR(95%CI)
**Maternal age at beginning of pregnancy**										
<25 years	107	50	9	1.00	1.00	69	81	16	1.00	1.00
25-34 years	117	107	36	1.42 (0.83-2.43)	2.21 (0.87-5.59)	90	137	33	0.65 (0.37- 1.14)	0.82 (0.32-2.09)
35+	41	51	19	1.31 (0.68-2.54)	2.20 (0.76-6.42)	22	77	12	1.60 (0.77-3.33)	1.49 (0.44-5.06)
**Maternal ethnicity**										
African American	165	71	21	1.00	1.00	137	98	22	1.00	1.00
Caucasian	85	122	39	2.00 (1.18-3.38)	2.99 (1.28- 7.00)	35	175	36	4.70 (2.60-8.51)	5.09 (2.07-12.49)
Asian/Native American	12	12	2	1.04 (0.39-2.80)	0.87 (0.15- 5.00)	8	16	2	1.10 (0.40-3.01)	0.77 (0.13-4.52)
**Maternal marital status**										
Married or live with partner	127	164	49	2.66 (1.59-4.45)	1.92 (0.86-4.33)	84	215	40	1.44 (0.87-2.41)	1.10 (0.46-2.69)
Not married or living with partner	137	45	15	1.00	1.00	97	80	21	1.00	1.00
**Maternal education**										
Less than high school	41	19	2	1.00	1.00	26	31	6	1.00	1.00
High school graduate/GED	97	39	10	1.12 (0.47-2.65)	3.40 (0.37-31.1)	78	61	7	0.85 (0.38-1.93)	0.62 (0.13-3.01)
College graduate/Graduate school Some college	126	151	52	1.70 (0.74-3.90)	7.60 (0.89-65.2)	77	203	48	1.69 (0.74-3.86)	2.37 (0.53-10.6)
**Second hand smoke**										
Yes	98	47	13	0.63 (0.36-1.12)	1.42 (0.57-3.50)	73	69	16	0.48 (0.27-0.84)	0.54 (0.21-1.38)
No	165	159	50	1.00	1.00	107	222	45	1.00	
**Parity**										
One	80	71	22	1.00	1.00	45	103	25	1.00	1.00
Two	51	46	16	1.02 (0.55-1.90)	1.63 (0.65-4.09)	36	63	14	0.90 (0.46-1.78)	0.73 (0.26-2.04)
Three or more	126	89	25	1.07 (0.62-1.85)	1.74 (0.69-4.04)	94	126	20	1.12 (0.61-2.04)	0.65 (0.26-1.62)
**Pre-pregnancy body mass index**										
<25 kg/m^2^	76	67	27	1.00	1.00	46	101	24	1.00	1.00
≥25 kg/m^2^	155	114	32	1.07 (0.67-1.71)	0.79 (0.39-1.59)	108	164	29	0.83 (0.50-1.39)	0.46 (0.20-1.05)
**Co-morbid conditions**										
Any chronic disease	105	77	13	0.83 (0.52-1.31)	0.45 (0.21-0.97)	70	101	24	0.92 (0.56-1.50)	0.95 (0.43-2.12)
None	159	131	51	1.00	1.00	110	194	37	1.00	1.00
**Cigarette smoking during pregnancy**										
Yes	69	36	7	0.82 (0.43-1.56)	0.40 (0.12-1.28)	36	64	13	2.37 (1.21-4.65)	1.39 (0.46-4.19)
No	195	173	57	1.00	1.00	145	231	48	1.00	1.00

*Odds ratios are mutually adjusted, referents are women reporting no supplement use

#### FA supplementation during pregnancy

Table 2 also shows that FA use exceeding 1,000 μg/d during pregnancy was associated with maternal age >35 years (OR = 2.4, 95%CI = 1.0-5.7), Caucasian race/ethnicity (OR = 6.4; 95%CI = 3.4-12.2), being married (OR = 2.2, 95%CI = 1.2-4.0), having a college or higher education (OR = 2.7, 95%CI = 1.0-7.0), being privately insured (OR = 3.0, 95%CI = 1.5-5.8), and having had three or more live births (OR = 0.4, 95%CI = 0.2-0.8). After simultaneous adjustment for each factor, being Caucasian (OR = 5.1, 95% = 2.1-12.5) remained associated with intake of FA doses exceeding the TUL. Interestingly, being Caucasian was also associated with FA use within recommended ranges (OR = 4.7, 95% = 2.6-8.5). Similar patterns of association emerged in smokers and in non-smokers.

Five percent of women reported FA supplementation ≥1,000 μg/d both before pregnancy and during pregnancy, and 75% were Caucasians with a college or higher education.

## Discussion

Our key findings were that overall, 51% of women reported some FA intake before pregnancy and 66% reported the same during pregnancy. In addition, more than 10% of women reported daily FA supplementation exceeding the TUL before or during pregnancy. Notably, 5% of the women reported taking FA exceeding the TUL, both before and during pregnancy. FA intake exceeding TUL was most frequently reported by Caucasian women, and least frequently by those reporting at least one morbid condition. Borderline associations were also found with advanced maternal age and having a college or higher education. Recently, data from the National Health and Nutrition Examination Survey estimated that in women of childbearing age, ~1% of women are exposed to FA doses exceeding 1,000 μg/d from fortified food alone, and 26% report FA intake in doses exceeding the recommended 400 μg/d [23].

Median folate intake among women of child-bearing age in the US is 450 μg/d (range 154-2,800ug/d) and approximately 75% is contributed by FA [10,23]. Because synthetic folate, or FA, is 70-85% bioavailable compared to 50% of folate occurring naturally in food [6], these findings suggest a sizable subpopulation of mothers and fetuses may be exposed to what some describe as supraphysiological folate levels [26]. To date, the adverse health effects of such exposure are unknown in humans. However, circulating folate levels have approximately doubled in the last decade [14,15]. Some large observational and randomized clinical trial data [27,28], but not all [29], suggest an increased risk of twin pregnancies in women with high circulating serum folate levels during pregnancy, independent of age and fertility. High FA intake early in pregnancy also has been linked to an increase in the frequency of the *methylenetetrahydrofolate reductase (MTHFR) *677T-allele in the fetus [30,31]. Carrying this genetic variant has been associated with chronic conditions including depression, schizophrenia, bipolar disorder, asthma, and wheezing later in life [32-35]. Conversely, peri-conceptional FA intake >400 μg/d was recently associated with improvements in cytosine-guanine (CpG) methylation at the *insulin-like growth factor *(*IGF2*) differentially methylated region (DMR) that regulates *IGF2 *imprinting in children [36]. Loss of imprinting at this *IGF2 *DMR has been associated with a higher risk of overgrowth disorders in childhood [37] and colon cancer in adulthood [38]. Our findings would suggest that a sizable proportion of pregnant women and their fetuses are exposed to FA exceeding the TUL. Because the original TUL was based on the potential for excessive folate to mask vitamin B12 deficiency (rare in pregnant women), and there are potential epigenetic benefits of FA supplementation, it is prudent to continue to monitor dosages of FA exposure in the population, and study its possible effects towards the goal of establishing limits based on the genotoxic and non-genotoxic effects.

The estimate of 66% FA use during pregnancy in our study is consistent with previous reports among pregnant women in the US [39,40] and other developed countries [29]. While the use of FA before pregnancy by 51% of women in the current study is higher than reports from previous studies of non-pregnant women in the US, which ranged from 27% in Arkansas to 44% in Rhode Island [39-44], it is similar to the 53% recently reported in an American six-site, hospital-based study [16]. The significance of the inverse association between morbidity and FA exceeding the TUL is unclear although it may reflect increased counseling of FA users since such conditions require frequent contact with health care providers. Associations between Caucasian race/ethnicity and high FA intake are consistent with previous reports where *any *FA supplementation was evaluated [39,45], and may reflect increased knowledge of B vitamin pharmacokinetics where excess intake may not be viewed as harmful. More probable is that Caucasian race/ethnicity and perhaps advanced maternal age and higher education represent greater wherewithal to access supplemental FA, suggesting a need for population-wide re-education on currently recommended FA use.

While the design is hospital-based, the distribution of demographic characteristics in this population is similar to that of the three-county region from which study participants largely arose [25,46] and are consistent with those reported by previous studies evaluating FA intake in the US and elsewhere [18,44,47,48]. A limitation of our findings is that we were unable to evaluate the potential effect of unplanned pregnancy in the association between potential correlates and FA supplementation. It is possible that FA intake exceeding TUL, during the prenatal period may be influenced by a desire to compensate for non-FA intake during peri-conception. The interpretation of these findings is also limited by our inability to prospectively monitor dosages of FA before and during pregnancy. Therefore, confirmation of these findings in larger studies will require active follow-up and validation of self-reports with serial measurement of maternal erythrocyte folate concentrations and dietary assessments. Meanwhile, prudence dictates that recommended FA doses are used by all women planning a pregnancy.

## Conclusions

In summary, while the FDA goal of consuming at least 400 μg/d of FA pre-conceptionally has been achieved by 50% of women of childbearing age, more than one-tenth of pregnant women consume daily doses that exceed the TUL with unknown effects to humans. The use of FA exceeding the TUL was associated with Caucasian race/ethnicity, advanced maternal age and a college or higher education. This study is part of a larger effort to characterize the role of early exposures on genetic and epigenetic perturbations in humans.

## Competing interests

The authors declare that they have no competing interests.

## Authors' contributions

CH conceived of the study, designed the study directed the data collection, analysis and interpretation of the data, and drafted the manuscript. APM oversaw participant recruitment in the clinic, contributed to interpretation of the results and drafting the manuscript. JMS contributed to the study design, analysis, interpretation of the data and drafting the manuscript. MRF contributed to analysis and interpretation of the data. BC performed the statistical analysis. WDW contributed to the inception of the research hypothesis and aims. JK contributed to the logistics of data collection and interpretation of results. RLJ contributed to analysis and interpretation of the data. SKM helped conceive the study, contributed to the data interpretation and drafting the manuscript.

All authors read and approved the final manuscript.

## Pre-publication history

The pre-publication history for this paper can be accessed here:

http://www.biomedcentral.com/1471-2458/11/46/prepub
